# Efficacy of frovatriptan in the acute treatment of menstrually related migraine: analysis of a double-blind, randomized, cross-over, multicenter, Italian, comparative study versus rizatriptan

**DOI:** 10.1007/s10194-011-0366-9

**Published:** 2011-08-13

**Authors:** Lidia Savi, Stefano Omboni, Carlo Lisotto, Giorgio Zanchin, Michel D. Ferrari, Dario Zava, Lorenzo Pinessi

**Affiliations:** 1 Neurologia II, Department of Neurology, Centro Cefalee, University of Torino, Via Cherasco 15, 10126 Turin, Italy; 2 Italian Institute of Telemedicine, Varese, Italy; 3 Ospedale Civile San Vito al Tagliamento, San Vito al Tagliamento, Italy; 4 Department of Neurology, University of Padova, Padova, Italy; 5 Leiden Centre for Translational Neuroscience, Department of Neurology, Leiden University Medical Centre, Leiden, The Netherlands; 6 Istituto Lusofarmaco d’Italia, Milan, Italy

**Keywords:** Migraine, Menstrually related migraine, Frovatriptan, Rizatriptan

## Abstract

The objectives of this study are to assess the efficacy and safety of frovatriptan, and rizatriptan in the subgroup of women with menstrually related migraine of a multicenter, randomized, double blind, cross-over study. Each patient received frovatriptan 2.5 mg or rizatriptan 10 mg in a randomized sequence: after treating 3 episodes of migraine in not more than 3 months with the first treatment, the patient had to switch to the other treatment. Menstrually related migraine was defined according to the criteria listed in the Appendix of the last IHS Classification of Headache disorders. 99 out of the 125 patients included in the intention-to-treat analysis of the main study were of a female gender: 93 had regular menstrual cycles and were, thus, included in this analysis. A total of 49 attacks classified as menstrually related migraine were treated with frovatriptan and 59 with rizatriptan. Rate of pain relief at 2 h was 58% for frovatriptan and 64% for rizatriptan (*p* = NS), while rate of pain free at 2 h was 31 and 34% (*p* = NS), respectively. At 24 h, 67 and 81% of frovatriptan-treated, and 61 and 74% of rizatriptan-treated patients were pain free and had pain relief, respectively (*p* = NS). Recurrence at 24 h was significantly (*p* < 0.01) lower with frovatriptan (10 vs. 32% rizatriptan). Frovatriptan was as effective as rizatriptan in the immediate treatment of menstrually related migraine attacks while showing a favorable sustained effect with a lower rate of migraine recurrence. These results need to be confirmed by randomized, double-blind, prospective, large clinical trials.

## Introduction

The high prevalence of migraine in the female population is well established: approximately one-quarter of women experience migraine at some time in their lives and over half of them report an association between menstruation and migraine [1]. Menstrually related migraine leads to substantial disability and attacks are more severe, longer in duration, and have a poorer response to analgesics than episodes occurring in other time of the month [2]. Drug prophylaxis may be useful for menstrual migraine [3].

Triptans have proved to be the most effective acute treatment of migraine headaches and are preferable for menstrual migraine in view of its difficult-to-treat nature [4, 5]. Prospective, randomized, controlled trials as well as retrospective analyses and open label studies support the use of triptans as acute therapy for menstrual migraine [5]. Sumatriptan, the first triptan to be marketed, has been shown to be well tolerated and effective in providing pain relief in menstrually associated migraine when administered in the mild pain phase, also in combination with analgesics [6–12]. Second generation triptans such as zolmitriptan [13–15], naratriptan [16], rizatriptan [17–21] and more recently almotriptan [14, 22] and frovatriptan [23] have been also successfully tested: the different pharmacokinetic and pharmacodynamic features of these triptans as respect to sumatriptan potentially render them particularly suitable for treating menstrual migraine. Following extensive evidence from prospective, double-blind, randomized, controlled studies, recent guidelines now recommend sumatriptan 50 and 100 mg or rizatriptan 10 mg for acute treatment of menstrually related migraine, and frovatriptan 2.5 mg or naratriptan 1 mg twice daily for preventive treatment of this condition [4, 24].

The preventive efficacy of frovatriptan has been largely demonstrated by randomized, double-blind, placebo controlled or open label studies [25–29]. Frovatriptan has been proved to be effective also in the acute management of menstrual migraine [30], including oral-contraceptive-induced menstrual migraine a subtype of menstrual migraine caused by estrogen withdrawal occurring during the week of pill suspension [31]. More recently, in an open-label post-marketing surveillance study conducted in women with migraine associated with menses, acute frovatriptan treatment improved patients’ ratings of treatment effectiveness and tolerability as compared to previously used triptans [32].

Since there are presently no studies comparing the efficacy of frovatriptan with that of another second generation triptan in menstrual migraineurs, an analysis of a recently published double-blind, randomized, cross-over study, comparing the efficacy of frovatriptan versus rizatriptan [33] has been carried out in a subgroup of women with menstrually related migraine and is reported in the present paper.

## Methods

### Study population

Subjects of both genders, aged 18–65 years, with a current history of migraine with or without aura, according to International Headache Society (IHS) 2004 criteria, and with at least one, but not more than 6 migraine attacks per month for 6 months prior to entering the study, were enrolled in the main study [33, 34]. In the present analysis, women with menstrually related migraine were selected. This condition was defined according to IHS criteria as migraine without aura attacks in a menstruating woman, occurring on day 1 ± 2 (namely days −2 to +3) of menstruation in at least 2 out of 3 menstrual cycles and additionally at other times of the cycle [34].

Main exclusion criteria were: (a) uncontrolled hypertension; (b) ischemic heart disease; (c) cardiac arrhythmias or symptomatic Wolff–Parkinson–White syndrome; (d) previous stroke or transient ischemic attack; (e) severe liver or renal impairment; (f) any other severe or disabling medical condition; (g) history of alcohol or analgesic or psychotropic drug abuse; (h) known hypersensitivity to study drugs; (i) previously demonstrated inadequate response to at least two triptans; (j) current use of propranolol or ergothamine (and its derivatives) as a prophylactic agent; (k) current use or use in the previous 2 weeks of MAO-inhibitors; (l) use of either test medication to treat any one of the last three episodes of migraine; and (m) other headaches that have been lasting for more than 6 days. Pregnant women and breast-feeding mothers were excluded as well, while women with childbearing potential but not practicing an effective method of birth control were to be submitted to a pregnancy test, if clinically indicated.

All patients gave written informed consent prior to their inclusion into the study. The study was approved by the Independent Institutional Review Boards of the study centers.

### Study design

Details on the study design are available in a previous publication [33]. Briefly, this was a multicenter, randomized, double blind, cross-over study, including 14 Italian centers (see Appendix). Each patient received frovatriptan 2.5 mg or rizatriptan 10 mg in a randomized sequence: after treating 3 episodes of migraine in not more than 3 months with the first treatment, the patient had to switch to the other treatment. Subjects were encouraged to treat 1 to 3 attacks for a maximum period of 6 months and to visit the center three times. Subjects having no migraine episodes during one of the two observation periods were excluded from the study.

During the randomization visit, after signing written informed consent, subjects provided a medical, treatment and migraine history. A physical and neurological examination and pregnancy test (if appropriate) were performed. Blood pressure and heart rate were measured for all the subjects. The degree of migraine-associated disability (MIDAS questionnaire) was also completed. At the end of the visit, a headache diary documenting characteristics of headache pain (including its relation with menses) and associated symptoms was dispensed with study medication. Subjects were instructed to treat at least 3 migraine episodes occurring in not more than 3 months and to come for the second visit. On this occasion, use of concomitant medications and occurrence of adverse events (from diary) were checked, blood pressure and heart rate were recorded, and a pregnancy test performed, if deemed necessary. The same procedures were carried out at the end of the second study treatment period or at the early withdrawal visit.

Patients were instructed to take one dose of study medication as early as possible after the onset of migraine attack. If insufficient relief had been obtained after 2 h, patients were allowed to take a second dose of study medication, with a maximum daily intake of two doses. In case of insufficient relief 1 h after the intake of the second dose of the study medication, patients were allowed to take a rescue medication. Alternate rescue medication could not include triptans, or contain ergotamine or its derivatives, or propranolol.

### Data analysis

This analysis was carried out in all normally menstruating women randomized to any of the two treatment sequences, having not positively refused to receive either study treatment and having treated at least one episode of menstrual migraine with both medications (intention-to-treat population).

The study endpoints were defined [34], as (a) the number of pain relief episodes at 2 h (a decrease in migraine intensity from severe or moderate to mild or none at 2 h); (b) the number of pain free episodes at 2 h (absence of migraine episodes at 2 h after intake of one dose of study drug); (c) the number of pain free episodes at 24 h (absence of migraine episodes at 24 h); (d) the number of pain relief episodes at 24 h (a decrease in migraine intensity from severe or moderate to mild or none at 24 h); and (e) recurrence within 24 h (episodes pain free at 2 h and headache of any severity returns within 24 h or intake of a second study drug or rescue medication). Consistency of response (responders in 2 and 3 out of 3 attacks) was also assessed for each study endpoint.

Changes in headache intensity from baseline after 2, 4, 24 and 48 h from study drug intake, were also evaluated. Analysis of pain free episodes was carried out also according to baseline headache intensity (mild, moderate or severe). Frequency of pain free and pain relief episodes was also assessed and compared between menstrually and non-menstrually related migraine attacks.

Continuous variables were summarized by computing average values and standard deviation (SD), while categorical variables by computing the absolute value and the frequency (as percentage). Endpoints were compared between groups by generalized estimating equation analysis. Kaplan–Meyer curves for cumulative hazard of recurrence over the 48 h were also drawn. *p* value refers to the statistical significance of between treatment difference. The level of statistical significance was kept at 0.05 throughout the whole study.

## Results

Overall 125 subjects formed the main study population [29]: 99 of them were of a female gender and 93 had a regular menstrual cycle and were, thus, included in this analysis.

Main demographic and clinical characteristics of the patients of the whole study population and of the subgroup of women with menstrually related migraine are reported in Table 1. As compared to the main study population, the women of this analysis were less tall (*p* < 0.01 between groups) and thinner (*p* < 0.01), and had a higher MIDAS score (*p* < 0.05). The proportion of patients suffering from attacks lasting more than 2 days was larger in the group of women with menstrual migraine (25 women vs. 1 woman with non-menstrual migraine, *p* < 0.05).

**Table 1:** Demographic and clinical baseline data of the 125 patients of the main study (33) and of the subgroup of 93 women with menstrually related migraine

	Main study *n* = 125	Menstruating Women *n* = 93	*p*
Age (years, means ± SD)	37 ± 9	36 ± 9	NS
Height (cm, means ± SD)	167 ± 9	163 ± 7	<0.01
Weight (kg, means ± SD)	64 ± 13	59 ± 8	<0.01
Age at onset of migraine (years, means ± SD)	16 ± 7	16 ± 6	NS
Migraine attack duration >2 days (*n*, %)	26 (21)	25 (28)	NS
MIDAS score (means ± SD)	22 ± 15	26 ± 17	<0.05

Data are shown as mean (±SD), or absolute (*n*) and relative frequency (%)

### Overall efficacy of study drugs

49 attacks (17% of all attacks) classified as menstrually related migraine were treated with frovatriptan and 59 (21%) with rizatriptan.

The proportion of pain relief episodes was similar (*p* = NS) between frovatriptan and rizatriptan at 2 h (58 vs. 64%) and at 24 h (81 vs. 74%). Also, the rate of pain free episodes at 2 and 24 h did not differ between the two treatment groups (31 and 67% frovatriptan vs. 34 and 61% rizatriptan; *p* = NS for both) (Table 2).

**Table 2:** Main study endpoints in the two study treatment groups

	Frovatriptan	Rizatriptan	*p*
Pain relief episodes at 2 h	25 (58)	27 (64)	NS
Pain free episodes at 2 h	15 (31)	20 (34)	NS
Pain relief episodes at 24 h	35 (81)	31 (74)	NS
Pain free episodes at 24 h	33 (67)	36 (61)	NS
Recurrent episodes at 24 h	5 (10)	19 (32)	<0.01

Data are reported as absolute (*n*) and relative (%) frequency. P refers to the statistical significance of the difference between the two study drugs

**Fig. 1:**
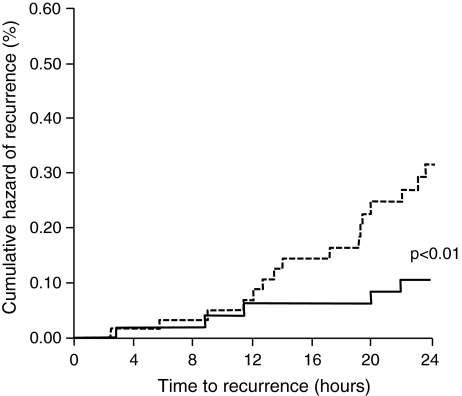
Cumulative hazard of recurrence over the 24 h during treatment with frovatriptan (*continuous line*) or rizatriptan (*dashed line*) in the 93 women with menstrually related migraine included in this analysis

The rate of migraine recurrence after 24 h was significantly (*p* < 0.01) lower under frovatriptan (10%) than under rizatriptan (32%). Also, the cumulative hazard of recurrence during the follow-up was significantly (*p* < 0.01) lower under frovatriptan (Fig. 1).

Consistency of response never resulted significantly different between the two groups, even for recurrences, probably due to the low number of subjects available for this sub-analysis (Table 3).

**Table 3:** Results of consistency analysis in the two study treatment groups

	Frovatriptan	Rizatriptan	*p*
Pain relief episodes at 2 h	19 (76)	20 (74)	NS
Pain free episodes at 2 h	11 (73)	16 (80)	NS
Pain relief episodes at 24 h	28 (80)	25 (81)	NS
Pain free episodes at 24 h	23 (70)	23 (64)	NS
Recurrent episodes at 24 h	3 (60)	16 (84)	NS

Consistency of was response was defined as responders in 2 and 3 out of 3 attacks. Data are reported as absolute (*n*) and relative (%) frequency. *p* refers to the statistical significance of the difference between the two study drugs

### Efficacy of study drugs according to baseline migraine intensity

Most of the treated attacks had a moderate or severe intensity at baseline. When rate of pain free episodes at 2 and 24 h was compared between treatments according to baseline migraine severity, frovatriptan was superior to rizatriptan at 2 h and at 24 h for treatment of moderate to severe attacks, with a statistically significant between treatment difference at 24 h (*p* < 0.01, Table 4).

**Table 4:** Absolute (*n*) and relative (%) frequency of pain free episodes at 2 and 24 h according to baseline headache intensity in the two treatment groups

	Frovatriptan	Rizatriptan		*p*
Pain free episodes at 2 hours				
Mild	3 (20)	8 (40)		NS
Moderate-severe	12 (80)	12 (60)		
Pain free episodes at 24 hours				
Mild	2 (6)	13 (24)		<0.01
Moderate-severe	31 (94)	23 (76)		

*p* refers to the statistical significance of the difference between the two study drugs

### Changes in migraine intensity

**Fig. 2:**
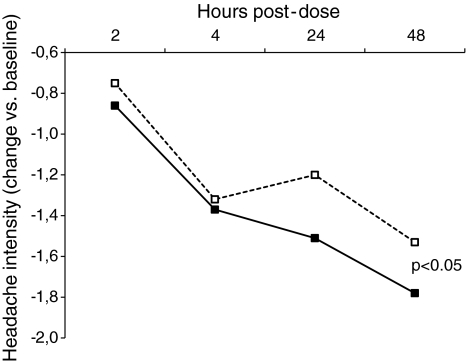
Changes in migraine intensity from baseline during treatment with frovatriptan (*continuous line*) or rizatriptan (*dashed line*) in the 93 women with menstrually related migraine included in this analysis

During the 48 h observation period, migraine intensity was progressively reduced by both drugs, but in a significantly larger degree with frovatriptan, particularly between 24 and 48 h (Fig. 2).

### Menstrually and non-menstrually related migraine attacks

Table 5 shows the number and proportion of pain free and pain relief-episodes at 2 and 24 h after frovatriptan intake for the 49 attacks related to and for the 233 attacks unrelated to the menstrual cycle. Frequency of pain relief at 2 and 24 h was 58 and 81% in menstrually related attacks and 54 and 87% in non-menstrually related attacks, with no statistically significant difference between the two types of migraine (*p* = NS). Similarly, the rate of pain free episodes at 2 and 24 h was 31 and 67% in menstrual migraine and 33 and 80% in non-menstrual migraine (*p* = NS).

**Table 5:** Absolute (*n*) and relative (%) frequency of pain free and pain relief episodes at 2 and 24 h in menstrually (*n* = 49) and non-menstrually related (*n* = 233) migraine attacks treated with frovatriptan. *p* refers to the statistical significance of the difference between the two types of migraine attacks

	Menstrually related migraine	Non-menstrually related migraine	*p*
Pain relief episodes at 2 h	25 (58)	100 (54)	NS
Pain free episodes at 2 h	15 (31)	77 (33)	NS
Pain relief episodes at 24 h	35 (81)	159 (87)	NS
Pain free episodes at 24 h	33 (67)	184 (80)	NS

## Discussion

In the present subgroup analysis of a double-blind, randomized, cross-over study, acute treatment of menstrually related migraine with frovatriptan and rizatriptan was associated with a similar immediate effect, as showed by similar proportions of pain relief and of pain free episodes at 2 and 24 h between the two drugs. However, frovatriptan showed significantly lower rates of headache recurrence over the 24 h than rizatriptan, this indicating a better sustained effect which can be explained by differences in pharmacological features of the two triptans. As a matter of fact, rizatriptan has a slightly shorter time to maximum concentration than frovatriptan, but the latter has a longer half-life (25–26 h vs. 2–3 h of rizatriptan), which might explain why frovatriptan, unlike rizatriptan, greatly reduced the risk of migraine recurrence [35–37].

The study provides also two important additional results, which are worthy being discussed. First, differences in drug efficacy were observed as a function of baseline migraine attack severity. In the women with migraine attacks of a moderate-severe intensity at baseline, the rate of pain free episodes at 24 h was significantly larger under frovatriptan. This is particularly relevant, considering that the subgroup of women with menstrually related migraine had a more disabling form of migraine as compared to the main study group, as confirmed by a significantly high MIDAS score and a larger proportion of patients with attacks of longer duration. Second, reduction in migraine intensity during the observation period was larger under frovatriptan, from 24 to 48 h after the onset of migraine attack and starting treatment, this confirming the more sustained pain relief effect of frovatriptan than rizatriptan.

This is the first study directly comparing the efficacy of frovatriptan as acute treatment of menstrually related migraine versus another triptan. Our study and a comparison study between almotriptan and rizatriptan [14], are the only two available head-to-head double-blind, randomized studies comparing the efficacy of two triptans in menstrual migraine. Though both studies share the limitations of subgroup analyses, they are useful because no such prospective studies on triptans in menstrual migraine have yet been carried out. Results of our study adds to the evidence of previous randomized, placebo controlled or open label studies proving the efficacy of frovatriptan as intermittent preventive [25–29] or acute treatment of menstrual migraine [30–32]. In published reports, prophylactic treatment with frovatriptan, started 2 days before the expected onset of headache and continued for 6 days, was always superior to placebo in reducing the frequency of menstrual migraine. A randomized, double-blind, placebo controlled study involving 410 women showed a headache incidence of only 8% when frovatriptan was given at dosage of 2.5 mg twice-daily and of 31% when given once-daily, while the incidence was 58% under placebo [26]. A post-hoc analysis of a randomized, double-blind, placebo controlled study in a population of 179 women experiencing menstrual migraine showed a significantly less prevalence of menstrual migraine with frovatriptan 2.5 mg twice (38%) or once-daily (51%) than with placebo (67%) [27]. These latter results are in line with those of a previous randomized, double-blind, placebo-controlled study of the same authors carried out in 546 women migraineurs [28]. Concerning treatment of the acute attack, in a 6-month open label study frovatriptan was given at 2.5 mg dose to 151 menstrual subjects for 2,439 migraine attacks, 659 of which occurring during a menstrual period [30]. Migraine relief within the 24 h was achieved for 82% of menstrual attacks, a proportion which was similar to that observed for non-menstrual attacks (87%). In an open-label uncontrolled study, including 20 women with a history of oral contraceptive-induced menstrual migraine, pain relief was reported in 55% of women 2 h after drug intake (58% in our study), while 10% of subjects were pain free after 2 h (31% in our study), 35% after 4 h and 60% after 24 h (67% in our study) [31]. Migraine intensity significantly decreased after 24 h by 1.6, an extent similar to that observed in our study at the same time point (1.5).

Evidence as acute treatment of menstrual migraine is available also for rizatriptan. In the published studies, rate of pain relief episodes at 2 h with rizatriptan was 64–78% (18–21), rate of pain free episodes at 2 h was 50% [20], while pain relief at 24 h was 63% [17] and pain free at 24 h was 32–34% [17, 20]: in our study, the corresponding figures for rizatriptan were very similar with respect to pain relief at 2 h (64%), slightly lower for pain free at 2 h (34%) and slightly higher for pain relief and pain free at 24 h (74 and 61%, respectively). No data on frequency of recurrences are available from previous studies on rizatriptan. Differences between our results and those of previous studies might be attributed to differences in study design and patients’ characteristics: in particular, in some heterogeneity in the definition of menstrual migraine is evident among studies.

## Conclusions

The present analysis of data from a multicenter, randomized, double-blind, head-to-head study suggests that frovatriptan and rizatriptan are similarly effective in the immediate treatment of women with menstrually related migraine. The study provides the first evidence that frovatriptan is superior to rizatriptan in reducing recurrence in menstrual migraine patients. The good sustained effect of frovatriptan shown in our study, seems to support indication of frovatriptan not only, as previously suggested, for preventive treatment of menstrual migraine [25–29], but also for managing the acute attack. The sustained analgesic effect of frovatriptan, supported by the lesser risk of recurrence may suggest the use of this drug for those patients with long-duration or recurrent migraine attacks [38, 39]. Caution must be taken when the drug is used within the same month for treating migraine attacks occurring during menses and outside the menstrual period, because of the risk of medication-overuse-headache, a risk which is, however, common to all triptans used for menstrual-migraine prophylaxis or treatment.

Given the retrospective design of our study, its results need to be confirmed by subsequent double-blind, randomized, prospective, large clinical trials.

## Appendix-list of study sites

*Coordinator* L. Pinessi (Torino).

*Investigators* P. De Martino (Torino), B. Panascia (Palermo), R. Rapisarda (Palermo), F. Devetag (Feltre), M.G. Sances (Pavia), L.A. Pini (Modena), G. Bono (Varese), R. Cerbo (Roma), M. De Marinis (Roma), M. Guidotti, R. Ravasio (Como), M. Alessandri (Grosseto), E. De Caro (Catanzaro), F. Lanaia (Catania), M.P. Prudenzano (Bari),G. Reggiardo (Biostatistical Unit, Mediservice, Milano), F. Sacchi (Clinical Unit, Mediservice, Milano).
